# Exogenous plant growth regulator and foliar fertilizers for phytoextraction of cadmium with *Boehmeria nivea* [L.] Gaudich from contaminated field soil

**DOI:** 10.1038/s41598-023-37971-8

**Published:** 2023-07-07

**Authors:** Wenxian Peng, Yejun He, Si He, Jinfeng Luo, Yi Zeng, Xiaoyang Zhang, Yingyi Huo, Yucheng Jie, Hucheng Xing

**Affiliations:** 1 Ramie Research Institute (Hunan Agricultural University), Changsha, 410128 China; 2 Key Laboratory of Germplasm Resources Innovation and Utilization, Changsha, 410128 China

**Keywords:** Plant sciences, Environmental sciences

## Abstract

As a enrichment plant, ramie can be used for the phytoremediation of cadmium (Cd)-contaminated soil. However, it is worth exploring the role of plant growth regulators and foliar fertilizers in the process of plant growth and development and Cd adsorption. By measuring the agronomic traits, Cd content of aboveground and underground ramie, calculating the Cd transfer coefficient (TF) and Cd bioconcentration factors (BCF), and the correlation between various indicators. This study examined the effects of plant growth regulators and foliar fertilizers on ramie’s capacity for Cd accumulation and transportation. Plant growth regulators and foliar fertilizers increased the Cd content of the aboveground ramie, reduced the Cd content of the underground ramie, and increased the TF. Among them, GA-1 increased the Cd content of the aboveground ramie to 3 times more than that of the control and reduced the Cd content of the underground ramie by 54.76%. Salicylic acid (SA) increased the Cd content of the aboveground ramie to three times more than that of the control. The combination of GA and foliar fertilizer reduced the Cd content of the aboveground and underground ramie and the TF and BCF of the underground ramie. After the hormones were sprayed, the TF of ramie had a significant positive correlation with the Cd content of the aboveground ramie; the BCF of the aboveground ramie had a significant positive correlation with the Cd content and TF of the aboveground ramie. The results indicate that Brassinolide (BR), gibberellin (GA), ethephon (ETH), polyamines (PAs), and salicylic acid (SA) have different effects on the enrichment and transport of Cd in ramie. This study provided an effective method to improve the capacity for ramie to adsorb heavy metals during cultivation.

## Introduction

With the advances in urbanization and industrialization, heavy metal pollution in China has become a serious problem. The pollution of water and soil poses a threat to the ecosystem, food security, agriculture, and sustainable development^1–3^. According to the Bulletin of the National Survey of Soil Pollution, 16.1% of the country’s soil contamination exceeded the legal limit, with heavy metal pollution contributing the most (82%)^4^. Cadmium (Cd) is a nonbiological essential heavy metal often combined with other heavy metals and one of the most toxic components of heavy metal pollution^5^. Cd-contaminated soil not only causes serious economic losses to agricultural production in China but also entails risks to human health. Therefore, research should be conducted on the prevention and control of Cd pollution in farmlands.

The phytoremediation of heavy metal-contaminated soils has received considerable attention^6^. The process involves using plants to remove pollutants from the environment^7,8^. Phytoremediation is a method of remediation that considers both ecological and economic effects and is a green technology developed for its strong potential to remove environmental pollution. Ramie (*Boehmeria nivea* [L.] Gaudich.) is the genus Urticaceae ramie's perennial bast fiber plant. It is commonly known as "China grass" and is widely cultivated for at least 5000 years in southern China^9^. Its fast growth, high fecundity, and high biological yield^10^ make up for the deficiencies of other hyperaccumulators, such as mustard plants^11^ and sunflowers^12^. Research has demonstrated that the bioavailability of Cd and lead in soil can be decreased through rhizosphere fixation and plant absorption and that soil can be stabilized by ramie and modifiers^13^. Although ramie is commonly used for its fiber, its products do not enter the food chain, and it is not associated with any health risk. Researchers have also modified varieties of ramie at the genetic level to improve its tolerance and ability to accumulate heavy metals^8^. Moreover, ramie is a permanent crop that provides ecological benefits to cultivation measures, and the cost of restoration can be recovered by ending continuous cropping. Therefore, ramie, the ideal phytoremediation material for Cd-contaminated soil, has great potential for use in the control of Cd pollution.

To obtain high removal efficiencies, lots of regulators including chelating agents and plant growth regulators have been used to improve the bioavailability of metals in soil and shoot biomass, respectively^14,15^. Plant growth regulators play a crucial role in the regulation of plant growth and development and in the response to external stresses^16^. The main Plant growth regulator are auxin, gibberellin (GA), cytokinin, abscisic acid, ethylene (ETH), and Brassinolide (BR) as well as some recently identified plant regulator, including polyamines (PAs) and salicylic acid (SA)^17^. GA has been proven to enhance the resistance of plants to heavy metal stress and to promote the accumulation of heavy metals^18^. Masood found that 10 mol L^−1^ GA can reverse the adverse effects of Cd on brassica. The 10^−6^ mol L^−1^ GA3 treatment increased Cd accumulation by 289% and the bioaccumulation coefficient by 128% in parthenium^10^. ETH is mainly used as a ripening agent in practical applications, but several studies have demonstrated that ETH plays a vital role in Cd stress. The tolerance of drupe to Cd can be increased by maintaining an appropriate level of ETH and a low ETH sensitivity through an antioxidant defense mechanism^19^. SA can reduce the accumulation of Cd in the aboveground part of rice^20^. SA can enable plants to resist abiotic stresses, such as ultraviolet radiation, low temperatures, heat shock, water deficit, salt injury, and heavy metals, and plays a role in the cross-protection response of plants to abiotic stresses^21,22^. SA can also increase mineral nutrition in plant organs and regulate the photosynthesis system, improving overall crop quality^23,24^. BR has been reported to promote plant growth, improve photosynthesis, and reduce heavy metal toxicity in plants^25,26^. PAs are compounds containing two or more amino groups. The raw materials used in its synthesis are ornithine and arginine. PAs play a crucial role in promoting the absorption of inorganic ions by roots, which improves resistance to stress and osmotic stress^27^. Binding PAs may play an essential role in resistance to Cu^2+^ stress^28^. Therefore, Plant growth regulators can improve the resistance of plants to heavy metals and promote the enrichment of heavy metals. Plant growth regulators can improve shoot biomass and enhance their accumulation capacity for heavy metals in aboveground plant parts. However, no effect of Plant growth regulators has been observed on the enrichment and transport of heavy metals in ramie.

Foliar fertilizer is a key source of nutrient elements for plant growth and development. Foliar fertilizer can also improve plants’ resistance to stress, promote plant growth and development, and increase yield. Potassium and phosphorous are the key elements for plant growth. Potassium activates many types of enzymes, which can enhance photosynthesis and the synthesis and metabolism of carbohydrates^29^. The use of potassium fertilizer during production can increase the yield and stress resistance of crops. Phosphorus is a key component of nucleic acid, nucleoproteins, phospholipids, and enzymes. The use of phosphorus fertilizer during production can enhance the crops’ resistance against drought and cold^30^. However, few studies have investigated the effects of fertilizers on the growth and ability of ramie to accumulate and transport Cd.

Plant growth regulators and foliar fertilizer can stimulate absorption of available nutrients that may be driven by the electrochemical gradient generated by the electrogenic H+-pump^31^. However, their activity depends on the concentration of their use, the environmental factors that affect their absorption, and the physiological state of the plant^15^. The effects of Plant growth regulators alone or in combination with Foliar fertilizer on the phytoextraction efficiency of ramie were unclear. In this study, In this study, two field experiment were conducted, one was using GA, ETH, SA, PAs, and BR foliar spray of ramie and another was using GA with KH2PO4 or KNO3 mixed foliar spray of ramie aimed to aimed to (1) investigate the treatment influence on Cd contents, translocation and accumulation in plant; and (2) estimate the treatment effects on the agronomic traits of ramie. These results will be helpful to compare the effects of different plant growth regulators and GA in combination with KH_2_PO_4_ or KNO_3_ as potential amendments for enhancing Cd phytoextraction by ramie.

## Materials and methods

### Plant materials and soil sample

Ramie is an asexual perennial plant propagated by using cuttings of lateral branches of approximately 15 cm in length. Ramie for Experiment A and Experiment B were arranged in two completely randomized plots with three replicates. Each plot contained six plants, planted in rows spaced 0.5 m apart, with a distance of 0.4 m between plants within rows. Experiment A: Ramie was planted in a Cd-polluted farmland in Hunan Agricultural University’s training base, Changsha City, Hunan Province, China. Experiment B: Ramie was planted in a Cd-polluted farmland in Liu yang City, Hunan Province, China. The lateral branches of ramie variety 171 were cut and propagated in April 2017. The plants were planted in the field in June 2017 and were mowed in December 2017. The ramie variety 171 was provided by the Ramie Research Institute of Hunan Agricultural University (Changsha, China).

Surface soil samples (0–20 cm) were taken from the test site, Then, the soil samples were air-dried and sieved through a 2-mm nylon screen to remove any debris before testing. Six air-dried soil samples were randomly taken to determine the physical–chemical properties (GB15618-2018). The soil type was red soil in both test sites, pH = 5.73 (Experiment A)and 5.78 (Experiment B), the average Cd content in soil was 3.27 mg kg^−1^ (Experiment A)and 3.43 mg kg^−1^(Experiment B), soil organic matter = 29.25 g kg^−1^ (Experiment A) and 21.54 g kg^−1^ (Experiment B), total nitrogen = 1.56 g kg^−1^ (Experiment A) and 1.13 g kg^−1^ (Experiment B), total phosphorus = 0.51 g kg^−1^ (Experiment A) and 0.43 g kg^−1^ (Experiment B), total potassium = 14.71 g kg^−1^ (Experiment A) and 11.56 g kg^−1^ (Experiment B).

### Field experiment

Experiment A and Experiment B were conducted at vigorous growing period. In experiment A, five plant growth regulators in different concentrations were sprayed into the positive and negative sides of ramie leaves in the corresponding plots. (Table 1) In experiment B, GA, KNO_3_ and KH_2_PO_4_ are compounded in different concentrations was sprayed onto the positive and negative sides of ramie leaves in the corresponding plots (Table 2). This procedure was repeated five times, each spraying amount is about 100 ml per plant, once every 15 days, from April 19, 2018. To reduce the effect of direct sunlight and to prevent the agents from being washed away by the rain, the agents were administered on sunny mornings when the sun is not too strong.

**Table 1:** Concentrations of different plant growth regulators.

Experiment	Treatments	Concentration
A	CK-1	0 mg L^−1^
	GA-1	50 mg L^−1^
	GA-2	100 mg L^−1^
	GA-3	200 mg L^−1^
	ETH-1	50 mg L^−1^
	ETH-2	100 mg L^−1^
	ETH-3	200 mg L^−1^
	SA-1	50 mg L^−1^
	SA-2	100 mg L^−1^
	SA-3	200 mg L^−1^
	PAs-1	0.1 mM
	PAs-2	1 mM
	PAs-3	10 mM
	BR-1	0.1 mg L^−1^
	BR-2	1 mg L^−1^
	BR-3	10 mg L^−1^

GA represents gibberellin, ETH represents eth-ylene, SA represents salicylic acid, PAs represents pol-yamine, BR represents Brassinolide, the number 1,2 and 3 indicates the increase of different treatment concentra-tions, respectively.

**Table 2:** Concentrations of different plant growth regulator and fertilizers.

Experiment	Treatments	concentration
B	CK-2	0 mg L^−1^ + 0%
	GW-1	50 mg L^−1^ + 0%
	GW-2	100 mg L^−1^ + 0%
	GW-3	200 mg L^−1^ + 0%
	GP-1	50 mg L^−1^ + 0.2%
	GP-2	100 mg L^−1^ + 0.4%
	GP-3	200 mg L^−1^ + 0.6%
	GN-1	50 mg L^−1^ + 1%
	GN-2	100 mg L^−1^ + 1.5%
	GN-3	200 mg L^−1^ + 2%

GW: GA + Water; GP: GA + KH_2_PO_4_; GN: GA + KNO_3_; The number 1,2 and 3 indicates the increase of different treatment concentrations, respectively.

The ramie grew in the soil for 125 days after transplantation and then harvested and divided into various parts for further processing. Before ramie was harvested, the agronomic traits of the ramie under each treatment were examined during the mature stage. Plant height was measured from the root neck to the upper most part of the stalk. The stem diameter (SD) and bark thickness (BT) were measured in the middle of stem using a Vernier caliper (ST22302, SG tools, Hangzhou, China). The area of the leaves was calculated by measuring their length and width with a straightedge. Plant biomass was measured by weighing both stems and leaves. Ramet number was measured through manual calculation. The tissues were carefully washed with tap water and double-distilled water to ensure no dust or other undesirable materials remained on the surface of the samples. The tissues were dried in an oven at 60 ± 5 °C for 4 days to ensure the constant weight. The weight of the samples was then measured, and the samples were crushed into powder for the Cd analysis.

Take the soil in the rhizosphere of ramie, and then the soil samples were air dried, crushed gently, and passed through a 2-mm sieve prior to the Cd analysis for calculation of index.

### Determination of Cd concentration

The dried plant materials and soil samples were ground into powder and sieved, and then digested in mixed acid with a HNO_3_-HClO_4_ solution^32^ and HCl-HNO_3_-HF-HClO_4_ solution^33^, respectively. The Cd concentration in the digested solution were determined using a graphite furnace atomic absorption spectrometer after a digestion (PinAAcle 900T AAS, Perkin Elmer Instruments Co., Ltd, Waltham, MA, USA). The linear fitting of the results of the samples measurements was 0.998 and the fitting degree of the equation was tested by chi square. Certified soil (GBW07405) and rice (GBW10045) reference materials were used for quality control, and the recovery rate of Cd was 89–102%.

### Statistical analysis

#### Comparison of Ramie field performance with different treatments

Field performance among different treatments were compared using ANOVA (analysis of variance) in SAS 9.4 software (SAS Institute, Cary, NC, United States). Plant data with different treatments were considered independent variables. The mean of each trait was tested at the p < 0.05 level and p < 0.01 level using Duncan’s multiple range test. (The following is the same) Evaluation of the overall field performance is a multi-criteria decision-making process that involves many factors.

In this study, a Membership function (MF) value and synthetic membership function (SMF) value were used to comprehensively express overall field performance^34^. The MF value of each field performance trait was calculated based on the following formula:

1 $$yi \; (k) = [xi \; (k)-\mathrm{min }x \; (k)] / [\mathrm{ max }x \; (k) -\mathrm{ min }x \; (k)]$$

where *yi*(k) represents the MF value of the k th field performance trait, *xi*(*k*) denotes the field-recorded value of the kth field performance, and max *x*(*k*) and min *x*(*k*) represent the largest and smallest value of *xi*(*k*), respectively.

The SMF value of each treatment was calculated based on the following formula:

2 $$\sum_{\mathrm{i}=1}^{\mathrm{i}=\mathrm{n}}yi(k)=[xi \; (k) -\mathrm{ min }x \; (k)] / [\mathrm{max }x \; (k) -\mathrm{ min }x \; (k)$$

#### Comparison of Cd related indexes of Ramie with different treatments

The accumulation and absorption of Cd in Ramie with different treatments can be shown by many indexes^35^. The (BCF) value of each treatment was calculated based on the following formula:

3 $$yBCF \;(k)= x \;\mathrm{ part \; of \; plant } \;(k) / x \; \mathrm{soil } \; (k)$$

where *yBCF* (*k*) the Cd bioconcentration factor value (BCF) of the k th treatments, *x* part of plant (*k*) denotes the Cd concentration value of the *k* th, and *x* soil (*k*) represent the Cd concentration value of the *k* th soil.

The Cd transfer coefficient (TF) value of each treatment was calculated based on the following formula:

4 $$yTF \;(k)= x \;\mathrm{ aboveground} \;(k)/x \;\mathrm{ underground} \;(k)$$

where *yTF*(*k*) represents the Cd transfer coefficient value (TF) of the k th treatments, *x* aboveground denotes the Cd concentration value of the *k* th aboveground, and *x* underground (*k*) represent the Cd concentration value of the *k* th soil.

The Enrichment quantity value of each treatment was calculated based on the following formula:

5 $$yEQ \;(k)=x \;\mathrm{ biomass} \;(k) * x \mathrm{Cd \; content }(\mathrm{aboveground}+\mathrm{ underground}) \;(k)$$

where *yEQ* (*k*) represents the enrichment quantity value of the *k* th treatments, *x* biomass(*k*) denotes the biomass of the k th treatments, and *x* Cd content (aboveground + underground) (*k*) represent the *k* th Cd content of the sum of aboveground and underground.

#### Correlation between Cd related indexes and agronomic traits of ramie in different treatments

Correlation analysis (CA analysis) was used to evaluate the relationship between the growth and development of ramie and the accumulation and absorption of Cd. Correlations between the ramie’s overall agronomic traits and the Cd related indexes were performed using the CORR procedure in SAS 9.4 software. Pearson’s correlation coefficients and their significance were used to assess the strength of the correlations.

All assays were made in triplicate. Graphs were drawn using GraphPad Prism 7.0(GraphPad Software, San Diego, CA, USA).

#### Legal statement

Experimental and field studies of plants (whether cultivated or wild) used in this study, including the collection of plant material, comply with relevant institutional, national and international guidelines and legislation.

## Results

### Analysis of agronomic traits and enrichment quantity after plant growth regulator treatment and mixture of GA and foliar fertilizers

#### Effects of plant growth regulators on agronomic traits and Cd enrichment

The effects of plant growth regulator on the agronomic traits were evident. Plant growth varied in accordance with variety and concentration of plant growth regulators. Plant height, stem diameter, skin thickness, and leaf area were the main parameters influencing biomass. As shown in Table 3, GA-3 and PAs-3 treatments significantly increased plant height. For all treatments, plant height significantly decreased after plants were sprayed with ETH. All hormone treatments except the ETH treatments caused significant increases in biomass accumulation compared with the control 1(CK-1) treatment. The effect of plant growth regulators treatments on the leaf area, stem diameter, and skin thickness of ramie was negligible; the ETH-3 treatment was the only treatment to significantly reduce these measures in comparison with control.

**Table 3:** Agronomic traits and Cd enrichment of ramie under different hormones.

Treatments	Plant height (cm)		Stem diameter (mm)		Skin thickness (mm)		Leaf area (cm^2^)		Biomass (kg ha^−1^)		Cd enrichment (mg ha^−1^)	
CK-1	212.07 ± 5.6DE		11.45 ± 0.58A		0.68 ± 0.01AB		228.93 ± 4.94AB		1659.13 ± 19.09I		22,709.46 ± 70.71 K	
BR-1	215.03 ± 2.87DE		11.02 ± 1.26A		0.68 ± 0.06AB		230.73 ± 1.37AB		2379.69 ± 72.11E		43,048.59 ± 173.46C	
BR-2	217.30 ± 5.28CDE		10.87 ± 0.70A		0.71 ± 0.10AB		225.86 ± 5.11AB		2378.18 ± 79.00E		23,480.56 ± 74.83 K	
BR-3	221.70 ± 5.86BCDE		11.64 ± 0.91A		0.74 ± 0.06AB		220.67 ± 13.82B		2339.15 ± 43.59EF		34,640.09 ± 71.18H	
ETH-1	145.30 ± 3.82F		12.94 ± 0.64A		0.68 ± 0.04AB		183.24 ± 8.37C		1025.15 ± 8.89 J		15,824.90 ± 142.77N	
ETH-2	132.07 ± 2.60G		12.39 ± 1.32A		0.75 ± 0.12AB		159.02 ± 5.07D		965.19 ± 25.98 J		14,297.68 ± 72.57O	
ETH-3	132.41 ± 3.58G		11.90 ± 1.06A		0.72 ± 0.08AB		128.65 ± 3.93E		1060.80 ± 88.88 J		17,588.06 ± 367.99 M	
GA-1	212.00 ± 3.00DE		12.10 ± 0.69A		0.73 ± 0.11AB		161.74 ± 3.78D		1859.60 ± 46.16H		28,856.40 ± 132.63 J	
GA-2	228.00 ± 4.35BC		12.02 ± 0.75A		0.84 ± 0.12A		195.03 ± 7.10C		2019.60 ± 20.52G		38,068.73 ± 500.58E	
GA-3	249.33 ± 4.62A		12.80 ± 0.60A		0.67 ± 0.04AB		200.62 ± 9.68C		2645.83 ± 109.34D		32,349.68 ± 146.97I	
PAs-1	219.73 ± 1.99BCDE		11.82 ± 1.19A		0.69 ± 0.06AB		223.83 ± 7.87AB		2568.07 ± 90.14D		40,748.15 ± 297.18D	
PAs-2	223.27 ± 1.16BCD		12.35 ± 1.26A		0.70 ± 0.02AB		218.02 ± 5.06B		3304.81 ± 86.69B		49,685.24 ± 362.86B	
PAs-3	241.87 ± 0.29A		11.67 ± 1.17A		0.83 ± 0.19A		227.30 ± 12.26AB		3980.73 ± 22.68A		58,875.00 ± 711.81A	
SA-1	209.80 ± 2.33E		11.43 ± 1.11A		0.69 ± 0.07AB		220.34 ± 3.94B		2209.42 ± 69.28F		36,772.11 ± 432.05F	
SA-2	220.27 ± 0.93BCDE		11.83 ± 1.50A		0.58 ± 0.09B		196.79 ± 6.54C		2254.69 ± 20.00EF		21,772.79 ± 285.77L	
SA-3	230.03 ± 13.83B		11.91 ± 1.71A		0.80 ± 0.17AB		239.35 ± 5.82A		2829.55 ± 54.44C		35,542.30 ± 216.07G	
Treatment	F	P	F	P	F	P	F	P	F	P	F	P
	164.90	***	0.82	0.65	1.35	0.23	56.18	***	515.02	***	3284.93	***

The same letters within a column indicate no significant differences (P > 0.01) among the treatments and CK-1. Values are means ± SD (n = 3). ***, P < 0.001; **, P < 0.01; *, P < 0.05., Duncan’s multiple range test.

Cd enrichment is the overall capacity of ramie to adsorb Cd. Treatment with PAs achieved the most noticeable effect on Cd enrichment; Cd enrichment after treatment with all three concentrations of PAs was significantly higher than after CK-1 treatment. Cd enrichment after treatment with SA-1 and SA-3 was significantly higher than that after CK-1 treatment. The BR-1 and GA-2 treatments also increased Cd enrichment. However, Cd enrichment after the ETH-1, ETH-2, ETH-3 treatments was lower than that after CK-1 treatment.

#### Effects of GA and foliar fertilizer mixture on agronomic traits and Cd enrichment

The GP and GN treatments positively affected the agronomic traits and Cd enrichment of ramie (Table 4). Plant height and biomass of ramie under GP-2, GP-3 and GN-2, GN-3 treatments were generally significantly higher than those under CK-2, but GP-1 and GN-1 treatments were significantly lower. Among the GW treatments, Cd enrichment after the GP-3 and GN-3 treatments was significantly higher than that after CK-2 treatment. The GP-1, GP-2, GP-3 and GN-1, GN-2, GN-3 reduced the biomass and Cd enrichment of ramie more than GW-1, GW-2, GW-3.

**Table 4:** Agronomic traits and Cd enrichment of ramie under different hormones and fertilizers.

Treatments	Plant height (cm)		Stem diameter (mm)		Skin thickness (mm)		Leaf area (cm^2^)		Biomass (kg ha^−1^)		Cd enrichment (mg ha^−1^)	
CK-2	183.8 ± 4.44D		10.25 ± 0.40AB		0.62 ± 0.03A		236.04 ± 3.72D		752.62 ± 12.30G		6894.00 ± 149.52E	
GP-1	171.2 ± 1.97E		8.73 ± 0.67B		0.59 ± 0.07A		201.55 ± 5.73E		1005.32 ± 6.65F		7144.47 ± 100.33E	
GP-2	219.3 ± 3.52B		11.48 ± 0.59A		0.74 ± 0.10A		258.05 ± 4.41C		1336.27 ± 6.81D		11,505.28 ± 818.62C	
GP-3	225.57 ± 4.55AB		12.28 ± 0.48A		0.66 ± 0.09A		272.58 ± 3.84AB		1449.54 ± 49.46C		15,198.43 ± 694.29B	
GN-1	182.68 ± 3.83D		10.38 ± 0.44AB		0.68 ± 0.10A		224.24 ± 6.61D		1153.65 ± 45.72E		8540.86 ± 224.84D	
GN-2	226.20 ± 5.34AB		12.37 ± 1.49A		0.73 ± 0.01A		265.71 ± 5.69BC		1398.06 ± 4.06CD		10,308.36 ± 617.67C	
GN-3	231.43 ± 3.19A		11.92 ± 0.39A		0.75 ± 0.09A		281.76 ± 7.62A		1456.82 ± 42.55C		15,689.95 ± 34.72B	
GW-1	206.67 ± 4.38C		12.01 ± 0.53A		0.7 ± 0.09A		275.8 ± 6.57AB		1629.20 ± 81.64B		15,444.82 ± 867.03B	
GW-2	189.03 ± 6.83D		11.92 ± 1.47A		0.7 ± 0.04A		256.23 ± 3.79C		2325.11 ± 49.98A		19,608.43 ± 48.11A	
GW-3	218.00 ± 2.62B		10.72 ± 0.68AB		0.56 ± 0.08A		275.14 ± 7.72AB		1626.20 ± 31.61B		14,240.09 ± 876.31B	
Treatment	F	P	F	P	F	P	F	P	F	P	F	P
	78.27	***	6.12	***	2.26	0.06	61.76	***	318.68	***	153.03	***

The same letters within a column indicate no significant differences (P > 0.01) among the treatments and CK. Values are means ± SD (n = 3). ***, P < 0.001; **, P < 0.01; *, P < 0.05, Duncan’s multiple range test.

### Effects of plant growth regulators on Cd content, Cd TF, and Cd BCF of ramie

#### Effects of plant growth regulators on Cd content of ramie

**Figure 1:**
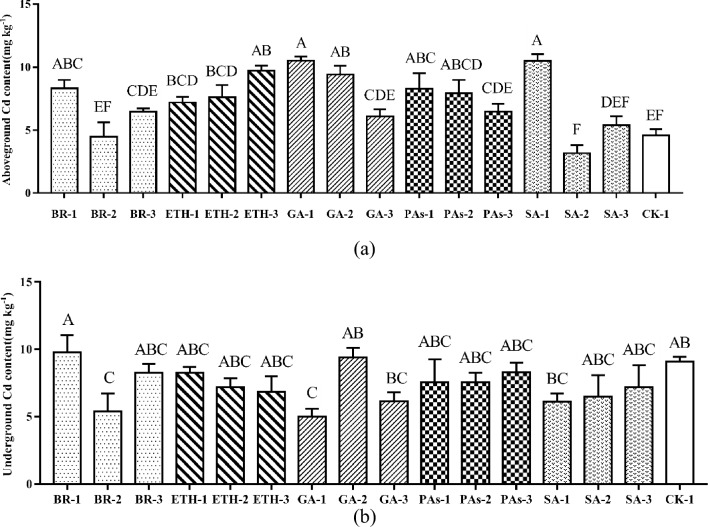
(**a**) Cd content in aboveground ramie sprayed by plant growth regulators; (**b**) Cd content in underground ramie sprayed by plant growth regulators; Bars marked with different letters are significantly different among treatments (P < 0.01). Values are means ± SD (n = 3). Duncan’s multiple range test.

Compared with the control, different plant growth regulators had different effects on Cd content in the aboveground parts of ramie. The Cd content of ramie aboveground varied depending on the type and concentration of plant growth regulators (Fig. 1a). The BR, GA, SA, ETH, and PAs plant growth regulator treatments increased the Cd content of the aboveground ramie. According to the results, the Cd content after treatments with various concentrations of plant growth regulators, except the BR-2,BR-3,GA-3, SA-2, and SA-3 treatments, was considerably higher than that of the control group. The GA-1 and SA-1 treatments exerted the strongest effect; Cd content after these treatments was 3 times higher than that after CK-1 treatment. The Cd content of the aboveground ramie in the GA-1, GA-2, GA-3 group and PAs-1, PAs-2, PAs-3 group decreased as the concentration of GA and PAs increased. The Cd content of the aboveground ramie in the ETH-1, ETH-2, ETH-3 group exhibited the opposite effect. The Cd content of the aboveground ramie in the SA-1, SA-2, SA-3 group was similar to that of the BR-1, BR-2, BR-3 group; as the concentration of SA and BR increased, the Cd content of the aboveground ramie decreased at first and then increased.

The plant growth regulators affected the Cd content of both the aboveground and the underground parts of ramie. The Cd content of the underground ramie after all treatments was generally lower than that of the control group (Fig. 1b), especially the Cd content of the groups treated with BR-2 and GA-1, which was 59.45% and 54.76% lower than that of the control, respectively. Similarly, the Cd content of the groups treated with GA-3, PAs-1, and PAs-2 was significantly lower than that of the control group. The Cd content of the underground ramie treated with BR decreased when the concentration of BR increased, which was contrary to the trend for the GA and SA treatments. The Cd content of the underground ramie after the ETH and PAs treatments did not change significantly.

#### Effects of plant growth regulators on Cd TF of ramie

**Figure 2:**
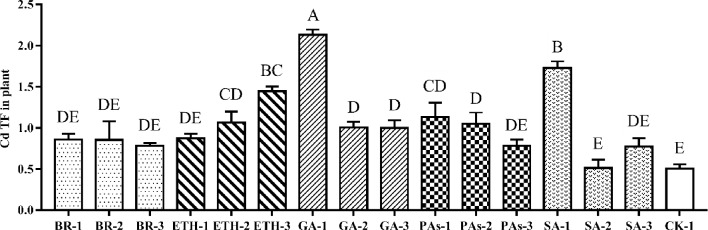
Cd translocation factors in aboveground ramie sprayed by plant growth regulators; Bars marked with different letters are significantly different among treatments (P < 0.01). Values are means ± SD (n = 3). Duncan’s multiple range test.

Cd TF refers to the ratio of Cd content of the aboveground part of ramie to that of the underground part. TF is an index used to evaluate the transportation of Cd from underground to aboveground parts of plants. Figure 2 shows that the Cd TF of ramie treated with lant growth regulators significantly increased. The TFs after the ETH-1, ETH-2, ETH-3 group treatment increased with an increasing concentration of ETH. By contrast, the TFs after the GA-1, GA-2, GA-3 group and PA-1, PA-2, PA-3 group treatment decreased as the concentration of the plant growth regulators increased. The TFs after the SA-1, SA-2, SA-3 group treatment decreased at first and then increased with the concentration of SA. The GA-1 treatment was the most effective and significantly stronger than CK-1, which yielded a TF greater than 2.

#### Effects of plant growth regulators on Cd BCF of ramie

**Figure 3:**
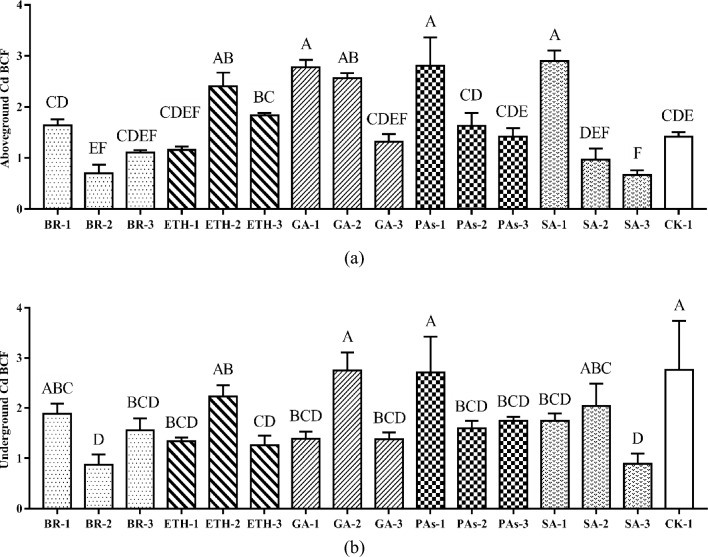
(**a**) Cd bioconcentration factors in aboveground ramie sprayed by plant growth regulators; (**b**) Cd bioconcentration factors in underground ramie sprayed by plant growth regulators; Bars marked with different letters are significantly different among treatments (P < 0.01). Values are means ± SD (n = 3). Duncan’s multiple range test.

A mount of heavy metals a plant absorbs and enriches from soil can be used as an indicator of the plant’s enrichment ability. The BCF of Cd is the ratio of the element content in a certain part of the plant to the corresponding element content in the soil. To a certain extent, the BCF of Cd reflects the degree of difficulty for an element to migrate through the soil–plant system and indicates Cd enrichment in plants. The inter-root soil Cd content and above-ground part Cd content were detected, and the BFC was calculated (Table S1). As shown in Fig. 3a, following GA-1, GA-2, GA-3, PA-1, PA-2, PA-3, and SA-1, SA-2, SA-3 treatments, Cd BCF decreased as GA, PAs, and SA concentrations rose. The Cd BCF treatment with GA-1, GA-2, PAs-1, and SA-1 were noticeably greater than the control group. Following SA-3 treatment, the aboveground ramie group's Cd BCF was significantly lower than that of the control group. The Cd BCF of aboveground ramie in the ETH-1, ETH-2, and ETH-3 groups grew initially before declining; the Cd BCF of aboveground ramie in the ETH-2 group was substantially greater than that in the CK-1 group.

The Cd BCF of the underground ramie after all plant growth regulator treatments was generally significantly lower than that after CK-1 treatment. The Cd BCF of the underground ramie after the GA-1 and SA-1, GA-3 and SA-3, PAs-2, and PAs-3 treatments were significantly lower than that after CK-1 treatment and did not markedly change after the GA-2, SA-3 and PAs-1 treatments. The changes in the Cd BCF of the underground ramie upon increasing concentrations of BR and ETH were similar to those in the aboveground Cd BCF (Fig. 3b).

### Effects of GA and foliar fertilizer mixture on Cd content, Cd TF, and Cd BCF of ramie

#### Effects of GA and foliar fertilizer mixture on Cd content of ramie

**Figure 4:**
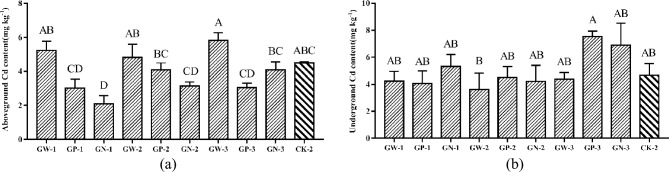
(**a**) Cd content in aboveground ramie by spraying mixed plant growth regulator and fertilizers; (**b**) Cd content in underground ramie by spraying mixed plant growth regulators and fertilizers; Bars marked with different letters are significantly different among treatments (P < 0.01). Values are means ± SD (n = 3). Duncan’s multiple range test.

GA significantly increased the Cd content, TF, and BCF of the aboveground ramie (Figs. 1, 2, 3). Because fertilizers composed of nitrogen and potassium are known to promote the growth and development of ramie, this study examined whether a mixture of nitrogen foliar fertilizer, potassium fertilizer, and GA could enhance the ability of ramie to absorb and enrich Cd. Figure 4a shows that treatment with a mixture of GA mixed and foliar fertilizers did not significantly affect the Cd content of the aboveground ramie compared with CK-2 treatment, except the GN-1 treatment, which reduced the Cd content by 59.88%. The combination of GA and foliar fertilizer significantly reduced the Cd content of the aboveground ramie in comparison with GA alone.

The combination of GA and foliar fertilizer did not significantly affect the Cd content of the underground ramie. The Cd content of the underground ramie after the GN-1, GP-3 and GN-3 treatments was slightly higher than that after CK-2 treatment (Fig. 4b).

#### Effects of GA and foliar fertilizer mixture on Cd TF of ramie

**Figure 5:**
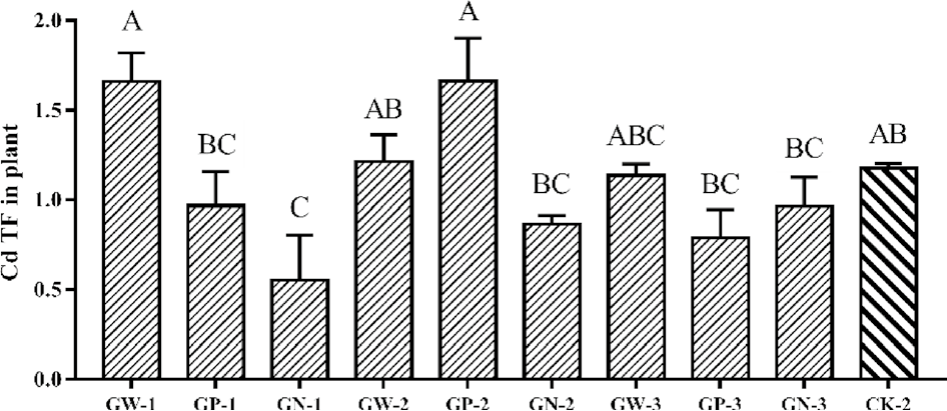
Translocation factors (TFs) from underground to aboveground treated by mixed plant growth regulator and fertilizers; Bars marked with different letters are significantly different among treatments (P < 0.01). Values are means ± SD (n = 3). Duncan’s multiple range test.

We discovered that unlike GA alone, the mixture of GA and fertilizers reduced the Cd TF of ramie. However, most compound treatments did not significantly affect the Cd TF in comparison with CK-2 treatment; only the GN-1 treatment significantly affected the Cd TF. The Cd TF after the GN-1 treatment was half that after CK-2 treatment (Fig. 5).

#### Effects of GA and foliar fertilizer mixture on Cd BCF of ramie

**Figure 6:**
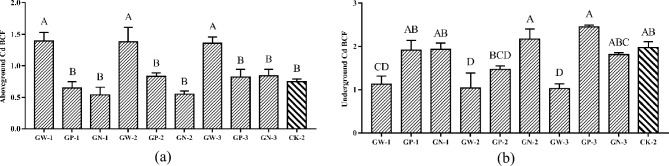
(**a**) Cd bioconcentration factors in aboveground ramie by mixed plant growth regulator and fertilizers; (**b**) Cd bioconcentration factors in underground ramie by spraying mixed plant growth regulator and fertilizers; Bars marked with different letters are significantly different among treatments (P < 0.01). Values are means ± SD (n = 3). Duncan’s multiple range test.

After treatment with Gw-1, Gw-2, and Gw-3, the enrichment coefficient of Cd in the aboveground part of ramie was much higher than that of the control group, as shown in Fig. 6a and Table S1. There were no statistically significant differences between the other therapies and CK. However, as shown in Fig. 6b, the Cd enrichment coefficient of ramie roots treated with Gw-1, Gw-2, and Gw-3 was much lower than that of the control group. Furthermore, the Cd enrichment coefficient of subterranean ramie in the GP-2 treatment group was marginally lower than in the control group. There was no statistically significant difference between the other therapies and CK.

The Cd BCF of the aboveground ramie after the GP-1, GP-2, GP-3 group treatment and GN-1, GN-2, GN-3 group treatment were generally lower than that after the CK-2 and GW-1, GW-2, GW-3 group treatment, and the interaction between GP-1, GP-2, GP-3 group treatment and GN-1, GN-2, GN-3 group treatment made the Cd BCF of the aboveground ramie significantly lower than did the GW-1, GW-2, GW-3 group treatment with the same concentration of GA (Fig. 6a).

The Cd BCF of the underground ramie after the GP-1, GP-2, GP-3 group treatment and GN-1, GN-2, GN-3 group treatment were generally higher than that after the GW-1, GW-2, GW-3 group treatment, but were not significantly different from that after CK-2 treatment, except for the GP-3 treatment (Fig. 6b). The Cd BCF of the underground ramie after the GP-2 treatment was not significantly different from that after the GW-2 treatment. Therefore, treatment with GA significantly reduces the Cd BCF of the underground part of ramie, but the mixture of GA of foliar fertilizer can negate this effect.

### Correlation analysis of traits

#### Effects of plant growth regulators on correlation

**Figure 7:**
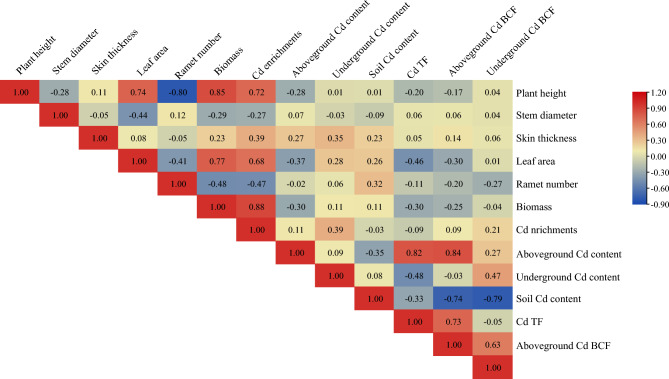
Correlation index of relevant indicators by plant growth regulators treatments. The shades of the colors and the corresponding numbers represent correlations. The darker the blue, the greater the negative correlation, and the darker the gray, the greater the positive correlation. Pearson’s correlation coefficients were used.

Figure 7 presents a significant correlation among various indicators after plant growth regulators treatment. For example, plant height was significantly correlated with leaf area and biomass. Leaf area and biomass were positively correlated with plant height, with correlation coefficients of 0.74 and 0.85 respectively. A significant positive correlation was observed between leaf area and biomass, with a correlation coefficient of 0.77. Aboveground Cd content was significantly correlated with Cd TF and aboveground Cd BCF. Cd TF and aboveground Cd BCF were positively correlated, with correlation coefficients of 0.82 and 0.84. A significant negative correlation was observed between soil Cd content, aboveground Cd BCF, and underground Cd BCF, with correlation coefficients of − 0.74 and − 0.79, respectively. The correlation coefficient of the significant positive correlation between Cd TF and aboveground Cd BCF was 0.73. The correlation coefficient of the significant positive correlation between aboveground Cd BCF and underground Cd BCF was 0.63. Cd enrichment had a significant positive correlation with plant height (0.72), leaf area (0.68), and biomass (0.88).

#### Effects of GA and foliar fertilizers mixture on correlation

**Figure 8:**
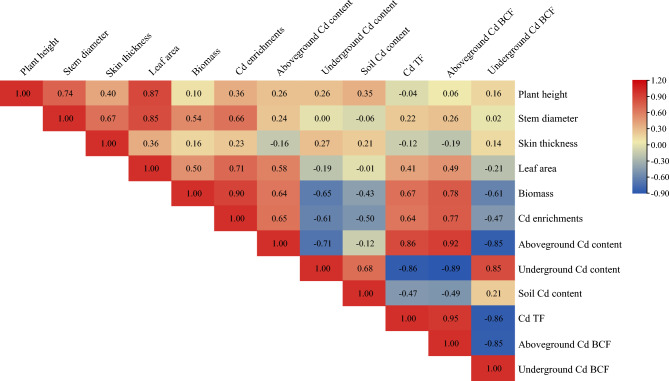
Correlation index of relevant indicators by mixed plant growth regulator and fertilizers. The shades of the colors and the corresponding numbers represent correlations. The darker the blue, the greater the negative correlation, and the darker the gray, the greater the positive correlation. Pearson’s correlation coefficients were used.

Biomass and Cd enrichments had a significantly positive correlation after treatment with GA and foliar fertilizer mixtures (Fig. 8). Aboveground Cd content was positively correlated with Cd TF (0.86) and aboveground Cd BCF (0.92) and negatively correlated with underground Cd BCF (− 0.85) and underground Cd content (− 0.71). A significant negative correlation was observed between soil Cd content and underground Cd content, with a correlation coefficient of − 0.68. Cd TF was significantly and positively correlated with aboveground Cd BCF and negatively correlated with underground Cd BCF, with correlation coefficients of 0.95 and − 0.86, respectively. Cd enrichment had a significant positive correlation with stem diameter (0.66), leaf area (0.71), and biomass (0.90).

## Discussion

Treating Cd soil pollution is an urgent task, and the phytoremediation technology-based approach can achieve superior results both economically and ecologically^36^. Ramie is a strong candidate and can promote the green revolution. Planting ramie can not only promote the development of the textile industry but also prevent soil pollution from entering the food chain, which would positively affect human health^37^. Investigation of the physiological and molecular mechanisms of Cd in ramie is crucial to regulating the amount of Cd moving from soil to plants and repairing soil. For the short-distance transport of Cd through the roots, phytochelatin secretion and vacuolar partition via ion channels and transporters are the key elements in the absorption, transport, and accumulation of Cd; for long-distance transport, the loading and unloading of phloem is a crucial element in the transport and accumulation of Cd to ramie plants. The transport and accumulation of Cd plants also causes physiological responses to Cd stress in ramie plants, which can manifest as changes in plant growth regulators levels, photosynthesis, water absorption, and mineral element absorption^38,39^. This study examined the effects of several plant growth regulators and the combination of fertilizers and GA on ramie. The increases in the Cd TF of ramie after the hormone treatments may be explained by the following: plant growth regulators caused the increases in the number of physiological and biochemical molecules absorbing and carrying Cd and enhanced the Cd resistance of ramie, resulting in the Cd enrichment of the aboveground part of ramie (Table 3).

GA, ETH, SA, BR, and PAs play a crucial role in alleviating abiotic stress and regulating the growth and development of plants^40,41^. According to results of this study, SA, BR, and PA played an essential role in the transportation of Cd in both the aboveground and underground parts of ramie, and the effects of BR and PA were dependent on concentration and ramie part (Table 3 and Fig. 2). GA is a plant growth stimulation hormone that regulates several physiological and biochemical processes, promotes growth and development, affects morphogenesis, and plays an essential role in the response to both biotic and abiotic stresses in plants^42,43^. Studies have demonstrated that plant growth regulators such as SA, GA, and indole-3-acetic acid (IAA) can alleviate abiotic stresses^44–47^. For example, the use of GA on leaves can reduce the uptake of nickel by mung bean plants, increase biomass, and promote growth. The importance of GA under abiotic stress has been well documented. This study confirmed that GA can promote the growth of ramie and improve its ability to absorb and enrich Cd (Table 3). ETH can inhibit the growth of plants, and the results in "Analysis of agronomic traits and enrichment quantity after plant growth regulator treatment and mixture of GA and foliar fertilizers" section indicated that ETH caused a decrease in biomass and Cd enrichment (Table 3). Studies on other plants have demonstrated that ETH can promote leaf abscission and plant maturation and inhibit apical dominance but that spraying at certain stages may produce the opposite effects^48^. The height of ramie decreased after ETH was applied, but the tillering and leaf area of ramie did not change significantly. ETH reduces the main components of biomass, thereby leading to a decrease of biomass. The enrichment of Cd in the plants decreased in accordance with changes in biomass (Table 3). BR are able to coordinate phytomorphogenesis, germination of seeds, cell division and elongation, flowering, vascular differentiation, formation of stomata, male fertility, and plant senescence^49^. BR, can increase the antioxidant enzyme content of plants and promote the secretion of heavy metal binding proteins, such as glutathione reductase (GR) and glutathione sulfotransferase (GST). This means that spraying plants with BR can increase their absorption of heavy metal ions^50^. In this experiment, spraying ramie plants with BR significantly increased their above-ground biomass and Cd enrichment (Table 3). Interestingly, the effect of low concentrations of BR was better than that of high concentrations. The effect of BR on Cd TF and BCF was minimal (Figs. 2, 3), suggesting that the Cd enrichment was mainly due to the increased plant biomass. SA participates in the coordination of plant growth and development, ripening, and responses to abiotic stresses^51^. Cd stress was found to increase the levels of free SA in the roots. This suggests that SA can reduce Cd toxicity by affecting Cd detoxification mechanisms, rather than activating antioxidant defense systems^52^. Additionally, SA can bind to JA and ethylene to increase plant transport. In this experiment, the low concentration of SA increased the biomass of ramie and significantly increased its Cd enrichment, aboveground Cd content, TF, and aboveground BCF (Table 3, Figs. 2, 3). It was hypothesized that SA increased the translocation capacity of ramie. Therefore, spraying ramie with SA may increase the above-ground cadmium accumulation in ramie. Polyamines (Pas) are nitrogen compounds present in all living cells. It participate in different cellular processes ranging from growth promotion and cell division to inhibition of ethylene production and senescence^53^. Many papers report changes in Pas levels in relation to heavy metal stress^54^. This experiment showed that polyamines significantly increased the height of ramie plants, the biomass, aboveground Cd content, and Cd enrichment (Table 3, Figs. 2, 3). It was hypothesized that polyamines could enhance the above-ground cadmium enrichment of ramie by increasing the plant height and biomass.

N, P, and K are vital nutrients for plant growth. The addition of fertilizer to the cultivation process benefits the growth and development of crops^55^. The results of this study demonstrated that GA alone or in combination with KNO_3_ or KH_2_PO_4_ positively influenced the agronomic traits of ramie (Table 4), indicating that the mixture of GA and fertilizer with N or P exhibited the same effects on the growth and development of ramie as in previous studies^56^. In addition, treatment with GP (including GP-1, GP-2, GP-3) and GN (including GN-1, GN-2, GN-3) promoted the enrichment of Cd in ramie (Fig. 6). This may be related to the physiological process ramie undergoes during heavy metal stress. A key regulator of plant growth and development, the functional site of GA at the cellular, tissue, and organ level of ramie is unknown. The site of application for GA on ramie can be a subject for future research. P^+^, K^+^, and Cd^+^ share the same transport pathway in plants. Although treatment with fertilizer promoted the growth and development of the plants, their Cd^+^ absorption and transport capacity decreased. Phosphates can increase the ionic strength of Cd adsorption^57^. Therefore, the decrease caused by GN (including GN-1, GN-2, GN-3) was more apparent than that caused by GP, especially in aboveground Cd content and aboveground BCF (Fig. 6). The BCF after the GP (including GP-1, GP-2, GP-3) and GN (including GN-1, GN-2, GN-3) treatments was lower than that after the GW-1, GW-2, GW-3 group treatment, which may be related to the chelation of root exudates (Fig. 6).

Plants’ response to Cd stress is a complex physiological process involving ion transporters^58^. Studies on yeast^59^, Arabidopsis^60^, and rice^61^ have demonstrated that adenosine triphosphate–binding cassette transporters, heavy metal–associated transporters, and natural resistance–associated macrophage protein transporters are involved in the response to Cd stress. The results of the study revealed common phenomena among the hormone treatments: the aboveground Cd content (Figs. 1 and 4) and Cd BCF increased (Figs. 3, 6); the underground Cd content and Cd BCF decreased (Figs. 1, 3, 4, 6); and the TF increased significantly after all hormone treatments (Fig. 2). Traditionally a textile crop, ramie has high cellulose, hemicellulose, and lignin content in the phloem, which establishes the conditions for Cd accumulation. The pulp inside ramie contains a large number of vessels that can transport Cd^+^. Hormones promote the growth of the aboveground part of ramie, the transport of Cd, and the accumulation of Cd in the underground part of the plant. Liu^62^ discovered that high concentrations of endogenous ETH delayed the formation of an ectoblast barrier and promoted the accumulation of Cd in the root ectoblasts. Studies by Neumann^63^ have demonstrated that ETH-mediated responses usually have high genotypic variability and may partially share common pathways under certain nutritional constraints. Although the biomass and Cd enrichment of ramie decreased after the ETH treatment, the Cd content and BCF of the aboveground ramie increased under high TFs (Table3, Fig. 2).

Phytoremediation of heavy metal-contaminated soil is a long-term process. Scientists face the challenge of how to overcome the problem of low biomass in cadmium-enriched plants, while high biomass plants are not tolerant to cadmium. Ramie, a crop with high cadmium tolerance, poses the question of how to improve its aboveground cadmium enrichment ability while maintaining its economic yield. Here, we propose using hormone treatment or hormone mixed with other leaf fertilizers to increase the cadmium concentration in the aboveground part of ramie, thus speeding up the process of phytoremediation. Our experiment showed that GA_3_ has a good effect. However, the use of GA_3_ will also reduce the fiber finesse of ramie^64^, which we want to avoid. In order to achieve sustainable development, ramie restoration of polluted land must make every effort to increase the accumulation of cadmium in the aboveground while ensuring economic benefits. The next step in the study of ramie remediation of heavy metal-contaminated soil can be explored through the mixed use of different hormones, foliar fertilizers, and soil fertilizers.

## Conclusions

This study concludes that spraying GA3, BR, SA, and PA on ramie leaves can increase the biomass and cadmium enrichment. This is mainly due to an increase in the cadmium TF and aboveground BCF. The use of ETH can also improve the TF and aboveground BCF, but it significantly reduces the biomass of ramie, leading to a decrease in cadmium enrichment. GA3 alone is more effective than the combination of GA3 and foliar fertilizer. However, the interaction between GA_3_ + KNO_3_ and GA_3_ + KH_2_PO_4_ can inhibit the absorption, transportation, and accumulation of cadmium. Therefore, planting ramie on polluted land and spraying plant hormones such as GA3, BR, SA, and PA can improve its ability to absorb and accumulate cadmium. Considering the price of drugs, it is recommended to use GA3 as the preferred hormone for improving cadmium remediation efficiency in crop ramie.

## Supplementary Information

Supplementary Information.

Supplementary Table S1.

## Data Availability

All data generated or analysed during this study are included in this published article [and its supplementary information files].
